# Is Uric Acid an Important Factor in Dental Disease?

**Published:** 1896-03

**Authors:** George D. B. Darby

**Affiliations:** Philadelphia


### IS URIC ACID AN IMPORTANT FACTOR IN DENTAL DISEASE?[1]

BY DR. GEORGE D. B. DARBY, PHILADELPHIA.

Mr. President and Members of the New York Odontological Society,—The title of my paper this evening is, "Is Uric Acid an Important Factor in Dental Disease?" The lithæmic or uric acid diathesis has engaged the attention of medical and dental scientists to a considerable extent during the past decade, and the physiological and pathological conditions which have been attributed to the presence of uric acid in the blood are almost legion. It is safe to assume that errors in diagnosis would reduce somewhat the number of diseases which the more zealous adherents of the theory ascribe to the retention of uric acid in the system. On the other hand, it is not unreasonable to suppose that a poison which is capable of producing such pathological conditions in one tissue or organ may not find its limitations until many organs and tissues have felt the effect of its baneful influence.

The presence of uric acid in the blood is not a new discovery, nor is it in small quantities attended with serious consequences.

Dr. Alexander Haig, of London, who has written quite extensively upon uric acid as a factor in the causation of disease, and who

---

[1] Read before the New York Odontological Society, November 19, 1895.

attributes many, if not all, of the diseases that the human body is heir to, to be due to uric acid directly or indirectly, says, in regard to his own case, "Having been all my life a sufferer from migraine, it was in the autumn of 1882 that, in despair of obtaining any complete relief from drugs, and not without some fear that I was really suffering from organic disease, I gave up all butcher meat and replaced it with milk and fish. A change was at once apparent, my headaches diminished both in frequency and severity, and from an average of one in a week they fell steadily, as the diet was persevered in, down to one in a month, one in three, six, or twelve months, and eventually eighteen months elapsed without an attack of notable severity. Having arrived, then, at the conclusion that leaving off butcher's meat had practically relieved me of headache, I began to ask why this was so, and at first I was inclined to attribute it to the formation of some poison, possibly of the nature of a ptomaine, in the intestines during the digestion of the butcher's meat. But a further study of the clinical history of migraine brought out such a strong relationship to gout that I began to suspect that uric acid might be the poison of which I was in search, and I therefore proceeded to estimate the excretion of uric acid and urea. At first I estimated only the excretion of twenty-four hours, and as many of my headaches lasted only a portion of a day, I got indefinite or contradictory results; but when I separated the urine excreted during the headache from that both before and after it, a definite and distinct relation between the headache and the excretion of uric acid at once became apparent,—a relation which I have since found in myself and others in very numerous instances, quite sufficient to remove the result out of the chapter of accidental coincidences. But once having noticed the relation of this headache to the excretion of uric acid, I soon noticed that each of its concomitant symptoms bore exactly the same relation to uric acid, that when the pulse was slow and of high tension there was always a greater excretion of uric acid than when it presented the opposite character, and the same with the mental depression and scanty urine. After this a little further experimentation brought out the fact that the excretion of uric acid was completely within my control, and that I could alter it from day to day or hour to hour in either direction at pleasure.

"I soon found out that in altering the uric acid I could alter the symptoms related to it; that when I produced an increased excretion with alkali, I produced the headache, mental depression, slow pulse, and scanty urine, and that when I stopped the plus excretion with an acid I removed all the symptoms; so that not only had I acquired the power to produce or remove the headache, but I had also the power to relax or contract the arterioles and capillaries to affect the tension of the pulse, the rate of the heart's action, and thus to influence the circulation in the brain, skin, kidneys, and probably the whole body. I also noticed that in curing a headache by giving an acid to diminish the excretion of uric acid, I always produced a certain amount of pricking and shooting pains in my joints, and it naturally occurred to me that the uric acid was held back in these joints and produced the pains. The uric acid that failed to appear in the urine must have gone somewhere. What more natural than to suppose that it had been retained in the joints, and that the pricking pains were the evidence of its presence?" Dr. Haig has found, after numerous experiments covering eight years, that a man of, say one hundred and fifty pounds, living upon a general diet, with meats, will form or introduce into his system some 18.1 grains of uric acid per day; but he will only excrete a part of this,—say 17.8 to 17.9 grains,—retaining in his system the remaining fractions of a grain, which, in a few years, will amount to many grains. He says, "I consider, therefore, that a man who eats what is called ordinary diet with butcher's meat twice a day, and does not take a great deal of exercise at the same time (for exercise keeps down the acidity and increases the excretion of uric acid), will, by the time he is thirty-five or forty, and certainly by the time he is fifty, have accumulated three hundred to four hundred grains of uric acid in his tissues, and possibly much more; and about this time, owing to the large amount of uric acid in his body, he will probably be subject to attacks of some form of gout or chronic rheumatism." It would seem as though this were particularly applicable to the troubles in the mouth with which we, as dentists, have to deal. For it is at or about this time of life that we find the first symptoms of pyorrhœa alveolaris and erosion appearing in the mouths of our patients, rarely before the thirtieth year. A number of cases that I have under my personal care are in the mouths of patients who have pronounced and well-defined gouty troubles, and others who, while they have no gout as yet, come of gouty ancestors, and who themselves are great meat-eaters and take little or no exercise. These facts would seem to bear out the assumption by Dr. Haig and others that a diet composed largely of nitrogenous foods was most favorable to the formation and retention of uric acid. Dr. Haig found, "with regard to the relation of uric acid to urea in excretion,—namely, 1 to 33 or 1 to 35,—other investigators

—Messrs. Yvon and Burlioz—found, as the result of one series of experiments, the relation of 1 to 30 and another 1 to 40; while Lecanu found much the same relation that I have,—viz., 1 to 33." Continuing, he says, "I have adopted the theory of Sir A. Harrod, that the final stage in the formation of uric acid is the formation of urate of ammonium in the kidney. According to this theory a large part of the urate so formed passes at once down the ureter and is excreted; but a small residue lingers in the kidney or the blood circulating in it, and is eventually carried over by the renal vein into the general circulation; when there it is, according to the same authority, attracted differently by different organs, and tends to be rendered less soluble, and so to be held back and accumulated in certain organs, as the liver, spleen, and certain fibrous tissues, especially those of joints, probably because these tissues are less alkaline than the rest of the tissues and fluids of the body."

Here, again, it would seem as though the teeth, covered, as they are, by a fibrous tissue and bathed almost constantly by an acid saliva, were fit subjects for the deposit of uric acid salts. Dr. Haig then says, "In the colder months of the year acidity on any given diet tends to be high (probably because there is little loss of acids in perspiration through the skin), and the balance of uric acid secretion tends to the side of retention or accumulation in the body; conversely, on the same diet, acidity rules low in the warmer months (owing to loss of acid in perspiration), and the excretion of uric acid exceeds its formation,—that is, more or less of the accumulations of the winter are washed out. This fact, that the excretion of uric acid is greater in the spring and summer months, has an important bearing on the seasonal variations of certain troubles that are due to uric acid. It is probable, however, that during the vigorous nutrition of adult life, up to and even past middle age, the acidity rules high, and year after year more uric acid is retained in the winter than is excreted in the summer, and the stores of urate in the body tend to increase; on the other hand, when from disease, or in the natural course of events from old age, nutrition begins to fail, acidity falls and remains low, excretion of urate remains more or less permanently above formation, as the urate accumulations of previous years are got again into solution in the blood and passed through the kidney into the urine."

To sum up, then, as to how uric acid is responsible for systemic disturbances in general, Dr. Haig says, "Uric acid acts as a factor in the causation of disease in one of two ways: first, as a direct local irritant when it is present in any tissues in considerable quantity and probably still in solution; second, as a contractor of arterioles and capillaries, affecting, on the one hand, the circulation and nutrition of all the organs and tissues of the body, and on the other producing high arterial tension which directly affects the heart and vessel walls and otherwise influences the intracranial and thoracic circulations."

So, much, then, for the formation and retention of uric acid and its action upon the general system. Dr. Haig has shown pretty clearly that it does exist and in comparatively large amounts in the blood and tissues of almost every one, and that from one cause or another it is quite sure to make its presence known when it has accumulated in sufficient quantity, which, he argues, is likely to occur at or about the thirty-fifth year.

The first mention of uric acid (that I am aware of) as a possible cause of trouble in the mouth was by Dr. Reese, of Galveston, Texas, in the discussion of a paper upon "Pyorrhœa Alveolaris," by Dr. Rawls, before the Southern Dental Association, in May, 1885, in which Dr. Reese gave it as his opinion that uric acid was responsible for true pyorrhœa alveolaris, and that he had had the deposit found upon the teeth of persons suffering from this disease examined by a chemist, and that it was pronounced to be composed largely of uric acid. The next year he read a paper, entitled "Uræmia and its Effects upon the Teeth," before the Southern Dental Association, in which he says, "In 1880 the writer became convinced that uric acid in the blood and saliva was the cause of grave trouble in the mouth." Again, he says, "One peculiarity of uric acid is that, while it will produce violent inflammation and intense pain, it rarely causes suppuration, except when in contact with the fluids of the mouth, and not always under these circumstances. When the saliva does not come in contact, and where it is protected from the air, we have no suppuration on the roots of the teeth. On the other hand, we very frequently meet, instead of an absorption, a bony deposit known as exostosis. You will sometimes observe the gum and alveolus absorbed from the palatal root of the superior molars without suppuration, and the labial roots apparently healthy. Extraction, however, reveals that almost always exostosis is present, especially if the tooth has no antagonist. The observation of the writer is that, while the formation of tophus upon the roots of the teeth is the usual concomitant of uric acid troubles, it is not necessarily so. The absorption of the peridental membrane may take place without any deposit whatever. The absorption takes place before any deposit occurs, and is always in advance of it, from a sixteenth to an eighth of an inch. I do not wish to be understood as saying that there is not a salivary calculus composed principally of phosphate of lime. You will sometimes see both this deposit and the tophus caused by uric acid on the same tooth, the phosphate being of lighter color and more porous."

It would seem, therefore, that this subject of uric acid as a cause of dental disease was thought of as long ago as 1880, and was made the subject of a paper in 1886, or nearly ten years ago. How much nearer the truth are we now than then? Pyorrhœa alveolaris and erosion have been made the subject of many papers and treatises during that time, being accounted for in many and various ways, but to my mind Professor C. N. Peirce has, in his paper on the "Etiology of Pyorrhœa Alveolaris," come nearer to solving the problem than any who have preceded him. He says, "In the first place I believe that, while pericementitis is associated with calcic deposit, the origin of the calcic salt and the antecedent condition which determines the locality and character of the deposit as well as the train of totally distinct symptoms which follow lead inevitably to the conclusion that two different diseases have thus far been confounded. In one form of pericementitis the origin of the calcic salt is the saliva, and in the other form, the blood. The former I shall designate as ptyalogenic calcic pericementitis, expressive of the idea that in its origin it is local, peripheral, and salivary. The latter I shall designate as hæmatogenic calcic pericementitis, expressive of the idea that in its origin it is constitutional, central, and associated with some modification of the normal composite of the blood plasma. That hæmatogenic calcic pericementitis is an altogether distinct affection from the preceding, and dependent for its cause upon some morbid material derived from the blood, will, I think, become apparent from the facts which I hope to adduce. Let it be understood that by this term reference is made to the disease with which we are all familiar as pyorrhœa alveolaris. The symptoms of this affection are too familiar to us all to detain us but a very few moments. Let it be recalled that in this form of the disease the morbid process begins on the root, and very frequently, if not usually, in the vicinity of the apical extremity, this being in marked contrast to the ptyalogenic form, which always has its origin at the gingival borders; that the inflammatory process is attended with pain, swelling, tissue disintegration, and the formation of pus; that there is a deposition of some insoluble salt upon the cementum, which, as it accumulates, eventually detaches the cemental membrane; that the alveolar process becomes absorbed; that the root of the tooth becomes horny in appearance, etc. These symptoms, taken in connection with the fact that thus far in the curative effort the disease has successfully resisted all forms of local treatment, whether manipulative or therapeutic, it seems, would lead us inevitably to the conclusion that we have here a distinct disease of constitutional origin. In ptyalogenic pericementitis it has long been recognized that the exciting cause is a deposition of calcic phosphate and carbonate around the gingival borders, which deposition infiltrating itself into the alveo-cemental membrane is the immediate cause of the subsequent inflammation and its concomitants. Without any apparent reason it has always been assumed that in the hæmatogenic form the cause was also the deposition of similar calcic salts. The fact, however, that in true pyorrhœa the symptoms are so different, and that all local treatment has been practically so unavailing, has suggested the possibility that some other chemical agent derived from the blood, and the product of some morbid constitutional state, might be the exciting cause." As Dr. Reese, had done, Professor Peirce also had the deposit taken from teeth that had been lost through pyorrhœa examined chemically, and found that this deposit was a combination of calcic urate, sodic urate, with some calcic phosphate and carbonate; showing beyond question that this deposit is a precipitate from the blood, and that the subsequent irritation is of constitutional origin, and that hæmatogenic calcic pericementitis, as Professor Peirce has chosen to call the disease we commonly know as pyorrhœa alveolaris, is undoubtedly due to the uric acid in the blood. Again quoting from Dr. Peirce's paper, he says, "The salts found in the exudation of gouty subjects are usually urates of lime, soda, and to some extent the phosphate and carbonate of lime. After the fluid has been exuded from the vessels, the non-appropriated lymph is absorbed by the lymphatics, and the salts left behind are deposited in both the amorphous and crystalline forms. The presence of the salts acting as foreign bodies excite the ordinary phenomena of inflammation. Let us now apply these facts to the disease in question,—viz., pyorrhœa alveolaris. Inasmuch as all portions of the body have been shown by pathologists to be liable to uric acid deposits, it is not at all strange that the alveola-cemental membrane, composed largely of connective tissue, should also become a depot for uric acid deposits. It is more than probable that as a predisposing cause there might coexist some impairment in the nutrition of this membrane dependent upon either local mechanical force or some obscure faulty innervation. However this may be, the mere presence of these salts leads to the conclusion that here as elsewhere they are derived from the blood by or through the medium of the lymph stream. With the absorption of the excess of lymph, the residual salts become precipitated upon the cemental surface. It is for this reason that I regard the deposition of uric acid as of blood origin, and the disease pyorrhœa alveolaris as one of the local manifestations of the constitutional state familiar to all pathologists as the uric acid or gouty diathesis."

As regards the constitutional treatment of these uric acid troubles in the mouth, I have recently talked with Dr. Peirce and Dr. E. C. Kirk, two firm believers in the efficacy of constitutional treatment, and equally firm in the belief that they cannot be cured nor even benefited permanently without such treatment; I have to report that Dr. Peirce has had and is having absolute cures from his form of treatment, which is as follows. Almost invariably patients suffering from pyorrhœa alveolaris will, upon inquiry, be found to be large feeders, and above all large meat-eaters, taking little or no exercise, frequently addicted more or less to the use of alcoholic liquors. In the first place all butcher's meat—such as beef, mutton, veal, etc.—is forbidden; in its place is substituted a diet of fish, the white meat of fowls, oysters, soft-boiled eggs, and milk; alcohol in every form is forbidden, and the patient is given the following treatment: fifteen minutes or half an hour before breakfast a glass of hot water with a five-grain tablet of tartarlithine dissolved therein, before luncheon another five-grain tablet in a glass of hot water, and after dinner the same dose is repeated, then at bedtime a glass of hot water without the tartarlithine is taken; so that the patient receives fifteen grains of tartarlithine and at least two quarts of hot water during the day. The tartarlithine dissolves the urates, and the hot water washes them out and aids their excretion in the urine. This treatment is kept up for several weeks, when the dose is gradually decreased, until but five grains of the tartarlithine are taken per day, but the hot water is kept up as before. Locally, any deposit there may be is carefully removed and the pockets treated with peroxide of hydrogen to remove any trace of pus there may be present, then washed out with a solution of hydronaphtol—one drachm to two ounces of water.

The patient is then given a prescription of hydronaphtol, ten grains; alcohol, one ounce; glycerin, one ounce, and water two ounces, and told to take a few drops and rinse the mouth several times a day. Under this treatment Dr. Peirce has had in every case marked and rapid improvement, and in several cases absolute recovery. Dr. Kirk's treatment is very similar to the above, except that he gives larger doses of the tartarlithine, in some cases as high as forty grains being given in the twenty-four hours; he also recommends in some cases the use of salicylate of ammonia, one drachm divided into six powders, taken three times a day; also tartarlithine and cascara in combination, and reports equally gratifying results as those treated by Dr. Pierce. It must be remembered that, as this is a constitutional disease, at least I firmly believe it to be, if the patient goes back to the former mode of living, with the use of butcher's meat, alcoholic drinks, lack of exercise, etc., the blood will again become charged with an excess of uric acid, the deposit will, in all probability, be again formed upon the teeth, with the subsequent formation of pus, etc., and all the symptoms of hæmatogenic calcic pericementitis be again present.

In this connection of uric acid in relation to dental disease, I believe, and I think those who have had more experience in its study and treatment than I will bear me out in the statement, that in many cases of pyorrhœa erosion is also present, to a greater or less degree, and from the same constitutional causes. In those cases where there is extreme wasting away of tooth-structure and perhaps no symptoms of pyorrhœa present, if the general health of the patient is inquired into, and his mode of living, it will be found that the symptoms are almost identical with those resulting from pyorrhœa, and that sooner or later the individual will be found to be a sufferer from rheumatism or gout, which are closely allied in the uric acid diathesis.

